# Development of a Human Intestinal Organoid Model for *In Vitro* Studies on Gut Inflammation and Fibrosis

**DOI:** 10.1155/2021/9929461

**Published:** 2021-07-27

**Authors:** Leonidas Kandilogiannakis, Eirini Filidou, Ioannis Drygiannakis, Gesthimani Tarapatzi, Stylianos Didaskalou, Maria Koffa, Konstantinos Arvanitidis, Giorgos Bamias, Vassilis Valatas, Vasilis Paspaliaris, George Kolios

**Affiliations:** ^1^Laboratory of Pharmacology, Faculty of Medicine, Democritus University of Thrace, Alexandroupolis, Greece; ^2^Gastroenterology and Hepatology Research Laboratory, Medical School, University of Crete, Heraklion, Greece; ^3^Laboratory of Molecular Cell Biology, Department of Molecular Biology and Genetics, Democritus University of Thrace, Alexandroupolis, Greece; ^4^GI-Unit, Third Department of Internal Medicine, National & Kapodistrian University of Athens, Sotiria Hospital, Athens, Greece; ^5^Tithon Biotech Inc., San Diego, USA

## Abstract

Inflammatory Bowel Diseases (IBDs) are characterized by chronic intestinal inflammation and fibrosis, the latter being the predominant denominator for long-term complications. Epithelial and mesenchymal 2D cultures are highly utilized *in vitro* models for the preclinical evaluation of anti-inflammatory and antifibrotic therapies. More recently, human intestinal organoids (HIOs), a new 3D *in vitro* model derived from pluripotent stem cells, have the advantage to closely resemble the architecture of the intestinal mucosa. However, the appropriate timing for the study of inflammatory and fibrotic responses, during HIO development, has not been adequately investigated. We developed HIOs from the human embryonic stem cell line, H1, and examined the expression of mesenchymal markers during their maturation process. We also investigated the effect of inflammatory stimuli on the expression of fibrotic and immunological mediators. Serial evaluation of the expression of mesenchymal and extracellular matrix (ECM) markers revealed that HIOs have an adequately developed mesenchymal component, which gradually declines through culture passages. Specifically, CD90, collagen type I, collagen type III, and fibronectin were highly expressed in early passages but gradually diminished in late passages. The proinflammatory cytokines IL-1*α* and TNF-*α* induced the mRNA expression of fibronectin, collagen types I and III, tissue factor (TF), and alpha-smooth muscle actin (*α*-SMA) primarily in early passages. Similarly, HIOs elicited strong mRNA and protein mesenchymal (CXCL10) and epithelial (CXCL1, CCL2, CXCL8, and CCL20) chemokine responses in early but not late passages. In contrast, the epithelial tight junction components, CLDN1 and JAMA, responded to inflammatory stimulation independently of the culture passage. Our findings indicate that this HIO model contains a functional mesenchymal component, during early passages, and underline the significance of the mesenchymal cells' fitness in inflammatory and fibrotic responses. Therefore, we propose that this model is suitable for the study of epithelial-mesenchymal interactions in early passages when the mesenchymal component is active.

## 1. Introduction

Inflammatory Bowel Diseases (IBDs), a group of diseases that includes Crohn's disease and ulcerative colitis, are characterized by chronic intestinal inflammation of unknown etiology [1]. Mucosal and systemic immunology has been the mainstream of IBD research for many decades resulting in the successful development of many biologics for the treatment of this debilitating group of diseases. However, epithelial and stromal biology has been largely overlooked. Recent studies have indicated that the study of the intestinal epithelium and mesenchyme may provide keys in deciphering the heterogeneity that characterizes patient phenotypes and their responses to biologics [2–4]. Furthermore, due to the fibrotic complications that eventually develop in more difficult to treat patients and the lack of therapeutic approaches to reverse postinflammatory fibrosis, the biology of the mucosal stroma has recently been brought into focus [4–6].

Significant progress has recently been achieved in understanding intestinal stromal cell biology by using 2D culture systems of primary mesenchymal cells isolated from human diseased and normal guts and intestinal organoids. During embryonic development and homeostasis, stromal cells have been shown to control epithelial proliferation and restitution through the production of activators and inhibitors of the Wnt signaling pathway [7]. During IBD-related chronic inflammation, we among others have shown that the intestinal stroma is not an innocent bystander, as previously thought [8–10]. Mesenchymal cells exhibit a variety of cytokine receptors and orchestrate extracellular matrix (ECM) production, accumulation, and eventually fibrosis in response to various inflammatory stimuli [8–10].

The development of human intestinal organoids (HIOs) in 2011 has revolutionized mucosal research as a novel *in vitro* system that enabled to study epithelial and mesenchymal cells as an interacting unit [11]. HIOs are 3D formations developed by pluripotent stem cells, through a process that simulates organogenesis. They have a similar architecture with the intestinal tissue, where the lumen is surrounded by epithelial cells forming villi and crypts, which are further supported by an outer layer of mesenchymal cells. Therefore, HIOs are able to approach intestinal inflammation and fibrosis in a more spherical way than classic 2D *in vitro* models, as they consist of many different interacting epithelial and mesenchymal cell types. HIOs still lack vascular, neurological, or immune structures, in comparison to animal models of IBD, but do provide a more analytical tool to separately study mesenchymal and epithelial biology from immune responses [12].

Despite their growing use for the study of monogenic diseases, intestinal organoids have rarely been used to model polygenic multifactorial diseases such as IBD. Recent studies have shown that as organoids are formed and later cultured, they continue to mature and change throughout their culture, mimicking the process of embryonic to fetal and adult development [13–15]. Therefore, knowing the appropriate time during their culture period to study inflammatory and fibrotic responses that mimic closely the IBD cascade is vital for these to be used as an effective *in vitro* disease model.

In this study, we successfully developed and characterized HIOs from the human embryonic stem cell line, H1. We examined the expression of fibrotic and mesenchymal factors during their maturation process, as well as the effect of the proinflammatory cytokines, IL-1*α* and TNF-*α*, on the expression of fibrotic and inflammatory mediators in HIOs during different stages of their maturation period.

## 2. Materials and Methods

### 2.1. H1 Cells

H1 cells are human pluripotent embryonic stem cells, originally derived and isolated from a male human blastocyst in 1998 [16]. They were purchased from WiCell (Madison, Wisconsin, USA) and set to culture according to WiCell Feeder Independent Pluripotent Stem Cell Protocols. Briefly, H1 cells were seeded onto Matrigel-coated 6-well plates (Matrigel™; Corning, New York, USA), which contained the mTeSR™1 medium (StemCell Technologies, Vancouver, Canada), and cultured in 5% CO_2_ at 37°C. H1 were fed daily and passaged every 5 days in a ratio of 1 : 6 using Dispase (MilliporeSigma, Burlington, Massachusetts, USA). Before passaging, H1 cells were first observed for any signs of differentiation, which can be visible when observed under a microscope, as differentiated cells significantly differ in morphology from undifferentiated embryonic stem cell colonies. According to the manufacturer's instructions, when the differentiation rate was above 5%, we removed the differentiated cells with a micropipette tip. H1 cells were maintained in culture and were regularly screened for the expression of pluripotent embryonic markers using immunofluorescence.

### 2.2. Development and Culture of HIOs

HIOs were developed from H1 embryonic stem cells using the STEMdiff™ Intestinal Organoid Kit (StemCell Technologies, Vancouver, Canada), according to the manufacturer's instructions. Briefly, H1 cells were seeded onto Matrigel-coated 24-well plates and cultured in the mTeSR™1 medium (StemCell Technologies, Vancouver, Canada) until they reached the appropriate confluency. H1 cells were then cultured in the Endoderm Basal medium containing Activin A and fed daily until day 3, when the Definitive Endoderm (DE) was created. DE was subsequently cultured in the Endoderm Basal medium containing Wnt3A and fibroblast growth factor 4 (FGF4) for another 5-6 days, until Mid-/Hindgut (MH) spheroids were released into the supernatant. MH spheroids were then collected, counted, seeded into domes made of Matrigel (Corning, New York, USA), and cultured in the Intestinal Organoid Basal (IOB) medium containing epidermal growth factor (EGF) and Noggin, until HIOs were finally formed. HIOs were continuously cultured in the EGF- and Noggin-supplemented IOB medium, fed every 3-4 days, and passaged every 10 days at a ratio of 1 : 3. HIOs and their intermediate developmental stages were characterized using immunofluorescence.

HIOs were cultured up to passage 13, and their intestinal structure proved stable until that passage (Supplementary Figure 1A). In addition, we semiquantitatively calculated the percentage of the organoid growth rate by measuring the diameter of three random organoids on day 0 and day 10 for each passage, which provides a semiquantitative estimate of their growth rate. As shown in Supplementary Figure 1B, the diameter increases by 50.8 ± 14.7% from day 0 to day 10 in passage 1, by 76.8 ± 11.4% in passage 6, and by 106 ± 5.8% in passage 13, suggesting that HIOs continue to mature their luminal structures even in late passages. In addition, we also performed double staining for the expression of Ki67, a well-known proliferation marker, and EpCam, an epithelial marker, in late-passage organoids and we found Ki67-positive expression in epithelial cells. This finding suggests that even in late passages, organoids continue to grow, and this growth is mainly attributed to the active proliferation state of epithelial cells. Supplementary Figure 1C is showing a late-passage organoid expressing Ki67 in its epithelial cells.

HIOs were cultured, and prior to cytokine stimulation, they were left with no growth factors for 15 h. Next, HIOs were stimulated with 5 ng/ml IL-1*α* and 50 ng/ml TNF-*α* for 12 h, and at the end of this incubation period, HIOs were collected for RNA extraction and mRNA expression analyses through qRT-PCR. In addition, we semiquantitatively calculated the percentage of organoid growth change by measuring the diameter of three random organoids in each time period and for each condition (control and 2C), in passages 2, 6, and 12.

### 2.3. Characterization of H1 Cells and HIO Development Using Immunofluorescence

H1 cells and HIO development were characterized using immunofluorescence, as previously described [9]. Briefly, samples were first fixed in 4% ice-cold paraformaldehyde (PFA; Sigma-Aldrich, St. Louis, Missouri, USA) for 40 minutes, then washed in phosphate-buffered saline (PBS; Sigma-Aldrich, St. Louis, Missouri, USA), and treated with 0.1% Triton X-100 (Sigma-Aldrich, St. Louis, Missouri, USA) for 15 minutes, in order to achieve membrane permeability. Samples were then treated with the blocking solution containing 5% bovine serum albumin (BSA; Sigma-Aldrich, St. Louis, Missouri, USA) for 1 hour and later incubated overnight at 4°C with primary antibodies in 0.5% BSA (Sigma-Aldrich, St. Louis, Missouri, USA). The next day, samples were washed and incubated with secondary fluorochrome-conjugated antibodies in 0.5% BSA (Sigma-Aldrich, St. Louis, Missouri, USA) for 2 hours. Finally, nuclei were stained either with DAPI (Sigma-Aldrich, St. Louis, Missouri, USA) and observed under a fluorescent microscope (Leica DM2000; Leica Microsystems GmbH, Germany) or with DRAQ5 (Novus Biologicals, Abingdon, UK) and observed in 3 dimensions under a light sheet fluorescent microscope (UltraMicroscope II; LaVision BioTec, Bielefeld, Germany).

In addition, we semiquantitatively calculated the percentage of vimentin-positive areas in passages 1, 5, and 10. In each passage, we measured the vimentin-positive area and compared it with the total organoid area, providing us with a semiquantitative estimate of the vimentin-positive area.

### 2.4. Light Sheet Microscope Setup and Imaging

The UltraMicroscope II (Bioimaging Facility, Department of Molecular Biology and Genetics, Democritus University of Thrace) is equipped with an Andor Neo 5.5 sCMOS camera (Andor Technology, Belfast, UK), with a pixel pitch of 6.5 *μ*m, a Nikon 16x (0.8 NA) water immersion objective, and a zoom body of 1.8x magnification, for a total of 28.8x magnification. The illumination is achieved by three intersecting light sheets coming from the right side, achieving a uniform illumination across the sample and reducing shadows and stripe artifacts. The detection axis is perpendicular and above the illumination path. The illumination NA was set to 0.156 creating a light sheet with a thickness of 2*w*_0_ = 4.53 *μ*m (as reported from the software; InSpector Pro). Excitation and detection were performed using a 488 nm, 561 nm, or 640 nm laser and 525/50 nm, 620/60 nm, and 680/30 nm filters, respectively. *z*-stacks were acquired with a 1 or 2 *μ*m step.

Fixed and stained HIOs were enclosed in the top surface of 1% low-melting agarose (in PBS) cubes and were immersed inside the imaging cuvette filled with distilled water. This technique ensures that the HIO structure remains undamaged and unpressurized, and therefore, the images taken depict their actual form. Image analysis, 3D rendering, and slice selection were performed in ImageJ (National Institutes of Health, USA).

### 2.5. Total RNA Extraction and Purification

Total RNA from HIOs was extracted and purified from genomic traces using the NucleoSpin RNA Plus XS kit (MACHEREY-NAGEL, Düren, Germany) according to the manufacturer's instructions. Briefly, HIOs were first lysed and homogenized, and DNA was removed by passing the lysate through the DNA removal columns. The purified lysate was then loaded onto the RNA extraction columns and washed 3 times, and finally, total RNA was eluted using RNase-free H_2_O. The concentration and purity of total RNA were measured using a Q5000 UV-Vis spectrophotometer (Quawell, San Jose, California, USA).

### 2.6. cDNA Synthesis and Quantitative Real-Time PCR

cDNA synthesis was performed using the PrimeScript RT Reagent Kit (Perfect Real Time) (TaKaRa, Kusatsu, Shiga, Japan) according to the manufacturer's instructions. In brief, 250 ng of total RNA was mixed with the 5X PrimeScript Buffer, reverse transcriptase, oligo dT primers, random hexamers, and RNase-free H_2_O and incubated at 37°C for 15 minutes. Reverse transcriptase was then inactivated by heat treatment. The gene-specific mRNA expression was quantified by quantitative real-time- (qRT-) PCR using the KAPA SYBR FAST qPCR Kit (Kapa Biosystems Ltd., Boston, MA, USA), as previously described [9]. Briefly, 25 ng of cDNA was mixed with the gene-specific primers, described in Table 1, and the KAPA SYBR FAST qPCR Master Mix, and a two-step amplification protocol was performed for almost all studied genes, except for tissue factor (TF), for which the annealing temperature was set at 52°C, and a three-step protocol was performed. All amplification reactions took place at a SaCycler-96 Real Time PCR system (Sacace Biotechnologies, Como, Italy), and the gene expression of each studied gene was normalized against GAPDH gene expression in the same sample using the 2^-*ΔΔ*Ct^ method. Regarding the results of the mesenchymal marker and ECM component expression through serial passages, passage 1 expression levels were set as a reference point and expression levels in later passages were compared to that.

### 2.7. Enzyme-Linked Immunosorbent Assay (ELISA)

Human DuoSet® ELISAs (R&D Systems, Minneapolis, Minnesota, USA) were used to estimate the protein concentrations of CCL2, CXCL10, CXCL1, CXCL8, CXCL10, and CXCL11 chemokines in HIO supernatants, according to the manufacturer's instructions. Briefly, flat 96-well plates were coated overnight with a capture antibody for each chemokine, and the following day, plates were incubated with the recommended blocking buffer for 2 h. Next, duplicates of each supernatant and known concentrations of chemokine samples were added in wells and incubated for 2 h, and then, a biotinylated detection antibody for each chemokine was added for another 2 h. Streptavidin-horseradish peroxidase was then added for 20 min, and the following addition of tetramethylbenzidine with H_2_O_2_ produced different optical densities (OD) of color which were measured at 450 nm on a microplate reader (DIAReader ELX800; DIALAB, Wr. Neudorf, Austria). The chemokine concentration was calculated using a linear standard curve according to the manufacturer's instructions.

### 2.8. Statistics

Results are presented as means with the standard error of the mean (SEM). Comparison of values among sample groups was performed with ordinary one-way ANOVA. Statistical significance was set at *p* < 0.05.

## 3. Results

### 3.1. Development and Characterization of HIOs

HIOs were developed from the embryonic stem cell line H1, as described in Materials and Methods. Prior to protocol initiation, the H1 pluripotent stem cell line was screened for embryonic stem cell marker expression, and it was found positive for Nanog, SOX2, and OCT4 (Supplementary Figure 1A). All major developmental stages of HIOs were assessed by relevant markers. The Definitive Endoderm (DE) was found positive for SOX17 and FOXA2, two transcription factors required for the development of the definitive gut endoderm and the intestinal tissue [17], respectively (Supplementary Figure 1B). Mid-/Hindgut (MH) spheroids were expressing CDX2, an intestinal epithelial marker [18], and vimentin and E-cadherin, mesenchymal and epithelial markers [6, 19], respectively (Supplementary Figure 1C), suggesting that the HIO formation were almost complete.

**Figure 1:**
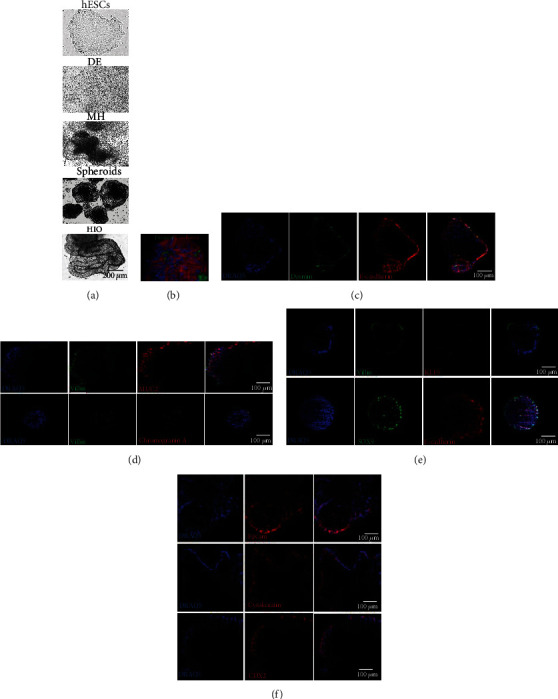
Development and characterization of HIOs. (a) Developmental stages of HIO formation. (b, c) HIOs stained against Desmin and E-cadherin, indicating fibroblast and epithelial cell populations, respectively. (d) HIOs stained positive for EpCam, cytokeratin, and CDX2, indicating intestinal epithelial cells. (e) MUC2-positive goblet cells and Chromogranin A-positive endocrine epithelial cells found in HIOs, surrounded by Villin-expressing epithelial cells. (f) HIOs stained positive for either KLF5 or SOX9, indicating the existence of intestinal epithelial stem cell niches that support the already-differentiated E-cadherin- and Villin-expressing epithelial cells. Representative 40x snapshots are shown in (a) and 28.8x immunofluorescence images in (b–f). (b–f) Images were obtained using a light sheet microscope. (c–f) Images are selected *z*-slices from the HIO total volume. (b) A 3D volume of an organoid.

After 9 days, HIOs were formed (Figure 1(a)) and were morphologically studied by immunofluorescence in order to confirm the presence of intestinal-specific cellular components. Developed organoids, as seen in Figures 1(b) and 1(c), included both the mesenchymal and epithelial cells, as indicated by positive immunoreactivity to Desmin and E-cadherin, respectively. The epithelium of HIOs consisted of intestinal CDX2-expressing epithelial cells (Figure 1(d)), forming a compact epithelial barrier, as they intensively expressed the cell adhesion molecules E-cadherin and EpCam (Figures 1(c) and 1(d)), which was further supported by abundant cytokeratin expression (Figure 1(d)). In addition, HIOs contained various types of epithelial cells, such as goblet (stained positive for MUC2, Figure 1(e)) and enteroendocrine cells (stained positive for Chromogranin A, Figure 1(e)), and formed villi as shown by their positivity for Villin (Figure 1(e)). Finally, SOX9 and KLF5 apparent staining revealed the concomitant presence of intestinal epithelial stem cell niches, possibly supporting the renewal of specialized epithelial cell subtypes.

### 3.2. The Mesenchymal Component Is Gradually Reduced upon Continuous Passaging

Previous studies have shown that organoids continue to mature and change throughout their culture. Since the presence of mesenchymal lineage cells is an essential difference in the cell components of embryonic stem cell-derived and adult stem cell-derived organoids, we decided to evaluate the persistence and functional fitness of mesenchymal cells during continuous passaging.

**Figure 2:**
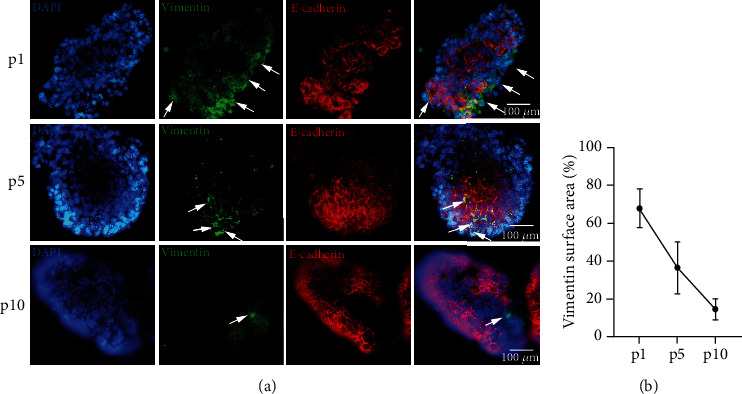
Mesenchymal evolution along HIO passaging. HIOs stained for the epithelial marker, E-cadherin, and the mesenchymal marker, vimentin, in three different passages (a). Vimentin-positive staining area is shown to be reduced through subsequent passages, suggesting that the mesenchymal component is gradually decreased (b). Representative 40x immunofluorescence snapshots are shown.

**Figure 3:**
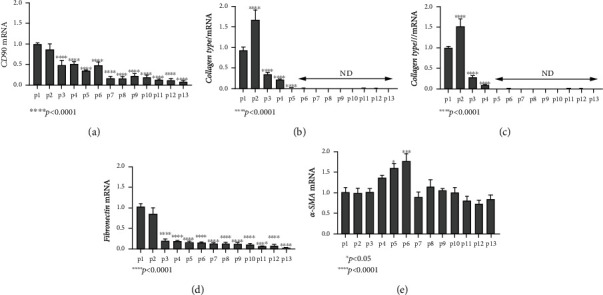
Expression of mesenchymal markers and ECM components during passaging. The mRNA levels of CD90, collagen types I and III, and fibronectin were gradually reduced after passage 2 (a–d), with the exception of *α*-SMA, which showed a tendency to increase during passages 4-6, but later decreased to basal levels (e). ND: nondetectable. All experiments were performed in triplicate. The gene expression of each studied gene was normalized against GAPDH gene expression in the same sample using the 2^-*ΔΔ*Ct^ method. Passage 1 expression levels were set as a reference point, and expression levels in later passages were compared to that. Data are presented as the mean ± standard error of the mean (SEM).

We therefore studied changes in the expression of vimentin and E-cadherin, two characteristic markers for mesenchymal and epithelial cells, respectively. Once fully developed, HIOs were maintained in culture and passaged every 10 days. At the end of each passage and prior to subculturing, a portion of HIOs was collected and stained using immunofluorescence. As seen in Figure 2, vimentin expression was affluent during the early passages but was later decreased. Indeed, semiquantitative calculation of the vimentin staining area in each passage revealed 68 ± 5.8% positivity in passage 1, 36.6 ± 7.9% in passage 5, and 14.6 ± 3.2% in passage 10 (Figure 2(b)), suggesting that the mesenchymal component was gradually reduced towards late passages. We further examined the mRNA expression of various fibrotic and mesenchymal factors, as organoids progressed through the passages. The mRNA levels of CD90, fibronectin, and collagen types I and III were gradually reduced after passage 2 (CD90: 0.08-fold, ±0.01, and *p* < 0.0001; fibronectin: 0.029-fold, ±0.003, and *p* < 0.0001; collagen type I: 0.0082-fold, ±0.0009, and *p* < 0.0001; and collagen type III: 0.0042-fold, ±0.0002, and *p* < 0.0001, Figures 3(a)–3(d)), with the exception of *α*-SMA, which showed more stable expression pattern during passages (Figure 3(e)).

### 3.3. The Effect of Proinflammatory Cytokines on the Expression of Fibrotic Mediators

We proceeded to study mesenchymal responses of HIOs to inflammatory stimuli in order to evaluate their suitability for modeling postinflammatory intestinal fibrosis. The effect of IL-1*α* and TNF-*α* on the expression of mesenchymal activation markers, ECM components, and profibrotic mediators was evaluated in passages 2, 4, 6, 8, 10, and 12. Prior to the experiments with IL-1*α* and TNF-*α*, we examined the expression of their receptors, IL1R1, IL1R2, and TNFRSF1A, and found that HIOs had a basal expression of all the receptors in all passages (data not shown). In order to exclude the possibility that IL-1*α* and TNF-*α* stimulation could affect the HIO structure and growth rate, we semiquantitatively calculated the percentage of organoid growth in passages 2, 6, and 12. Supplementary Figure 3 depicts the controls or 2C-treated HIOs before and after all incubation periods, along with the percentage of diameter changes during these incubations, in three representative passages (2, 6, and 12). As shown in Supplementary Figure 3B, D, and E, the percentages of diameter changes are negligible among the passages and conditions. In passage 2, the diameter changes in controls are 2.2 ± 1.4% at 12 h, 5.5 ± 0.4% at 24 h, and 8.2 ± 1.7% at 48 h and in 2C 1.8 ± 0.8% at 12 h, 1.8 ± 0.5% at 24 h, and 8.1 ± 1.7% at 48 h. In passage 6, the diameter changes in controls are 4.8 ± 1.1% at 12 h, 3.1 ± 1.1% at 24 h, and 4.5 ± 0.6% at 48 h and in 2C 1.7 ± 0.8% at 12 h, 6.4 ± 1.3% at 24 h, and 3.3 ± 0.6% at 48 h. In passage 12, the diameter changes in controls are 4.2 ± 1.9% at 12 h, 2.5 ± 1.3% at 24 h, and 5 ± 0.9% at 48 h and in 2C 3.3 ± 0.7% at 12 h, 3.6 ± 0.6% at 24 h, and 6.3 ± 1.1% at 48 h.

**Figure 4:**
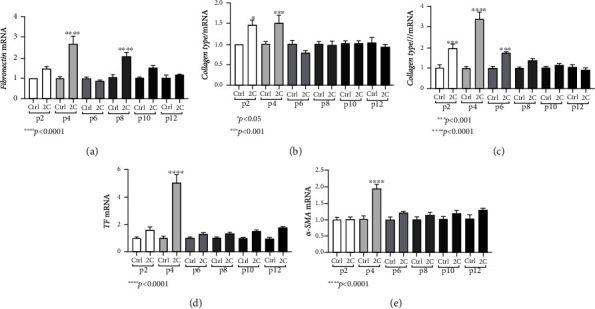
HIOs exhibit fibrotic responses to inflammatory cytokines. IL-1*α* and TNF-*α* (2C) induced the mRNA expression of *α*-SMA in passage 4 (a), fibronectin in passages 4 and 8 (b), TF in passage 4 (c), collagen type I in passages 2 and 4 (d), and collagen type III in passages 2, 4, and 6 (e). Concentrations of cytokines used: IL-1*α* 5 ng/ml, TNF-*α* 50 ng/ml. All experiments were performed in triplicate. The gene expression of each studied gene was normalized against GAPDH gene expression in the same sample using the 2^-*ΔΔ*Ct^ method. In every passage, expression levels of treated organoids were normalized against those of the control ones. Data are presented as the mean ± standard error of the mean (SEM).

We observed a differential response of HIOs to the inflammatory stimuli depending on the passage. Specifically, IL-1*α* and TNF-*α* induced a statistically significant upregulation of ECM components such as collagen types I and III and fibronectin in early passages with maximum responses observed in passage 4 (fibronectin: 2.69-fold, ±0.87, and *p* < 0.0001; collagen type I: 1.52-fold, ±0.17, and *p* < 0.001; and collagen type III: 3.39-fold, ±0.32, and *p* < 0.0001, Figures 4(a)–4(c)). Likewise, maximum responses of the profibrotic mediator TF and the mesenchymal activation marker *α*-SMA also occurred in early passages and specifically in passage 4 (TF: 5.04-fold, ±0.59, and *p* < 0.0001; *α*-SMA: 1.95-fold, ±0.12, and *p* < 0.0001, Figures 4(d) and 4(e)). Interestingly, fibrotic mesenchymal responses to proinflammatory cytokines were gradually reduced in later passages and eventually diminished in passage 12 (Figure 4).

### 3.4. The Effect of Proinflammatory Cytokines on the Expression of Mesenchymal and Epithelial Inflammatory Responses

We next proceeded in investigating the effect of the proinflammatory cytokines, IL-1*α* and TNF-*α*, on the mesenchymal and epithelial inflammatory responses of HIOs.

**Figure 5:**
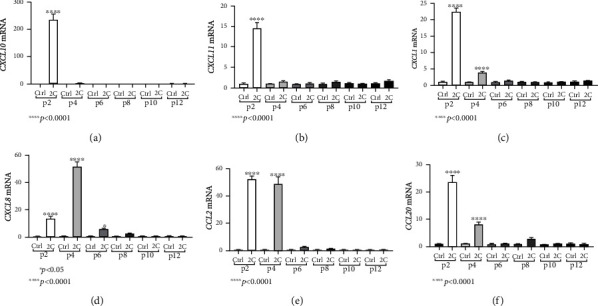
HIOs exhibit mesenchymal and epithelial chemokine mRNA responses to inflammatory cytokines. IL-1*α* and TNF-*α* (2C) induced the mRNA expression of CXCL10 in passage 2 (a), CXCL11 in passage 2 (b), CXCL1 in passages 2 and 4 (c), CXCL8 in passages 2, 4, and 6 (d), CCL2 in passages 2 and 4 (e), and CCL20 in passages 2 and 4 (f). Concentrations of cytokines used: IL-1*α* 5 ng/ml, TNF-*α* 50 ng/ml. All experiments were performed in triplicate. The gene expression of each studied gene was normalized against GAPDH gene expression in the same sample using the 2^-*ΔΔ*Ct^ method. In every passage, expression levels of treated organoids were normalized against those of the control ones. Data are presented as the mean ± standard error of the mean (SEM).

Similar to fibrotic mesenchymal responses, the chemokine responses of the HIO mesenchyme to inflammatory stimuli were strong during early passages but diminished in later passages. Specifically, IL-1*α* and TNF-*α* stimulation induced a statistically significant upregulation of CXCL10 and CXCL11 mRNA levels in passage 2 (CXCL10: 235.3-fold, ±20.73, and *p* < 0.0001; CXCL11: 14.53-fold, ±1.28, and *p* < 0.0001) but had no effect later on (Figures 5(a) and 5(b)).

As for the epithelial inflammatory responses of HIOs, we chose to study the effect of IL-1*α* and TNF-*α* on the expression of chemokines that are mainly produced by epithelial cells and on tight junctions, which characterize the epithelial component. Again, the effect of IL-1*α* and TNF-*α* was different depending on the passage.

Regarding the chemokine expression, in passage 2, IL-1*α* and TNF-*α* stimulation led to a statistically significant upregulation of all studied chemokines (CXCL1: 22.30-fold, ±1.30, and *p* < 0.0001; CXCL8: 13.30-fold, ±1.76, and *p* < 0.001; CCL2: 52.29-fold, ±2.59, and *p* < 0.0001; and CCL20: 23.85-fold, ±2.43, and *p* < 0.0001, Figures 5(c)–5(f)). In passage 4, the effect of IL-1*α* and TNF-*α* was even more intense for CXCL8, as it was even greater upregulated (51.98-fold, ±3.40, and *p* < 0.0001, Figure 5(d)), remained the same for CCL2 (48.91-fold, ±5.28, and *p* < 0.0001, Figure 5(e)), and was weaker for CXCL1 and CCL20, as their mRNA expression, although upregulated when compared to unstimulated organoids, was lower than passage 2 (CXCL1: 3.89-fold, ±0.22, and *p* < 0.0001; CCL20: 8.12-fold, ±0.87, and *p* < 0.0001, Figures 5(c) and 5(e)). In passage 6, only CXCL8 remained upregulated in response to proinflammatory cytokines, although its expression was significantly lower than that of passage 4 (5.76-fold, ±0.33, and *p* < 0.05, Figure 5(d)). As for passages 8, 10, and 12, none of the studied chemokines was increased in response to IL-1*α* and TNF-*α*.

**Figure 6:**
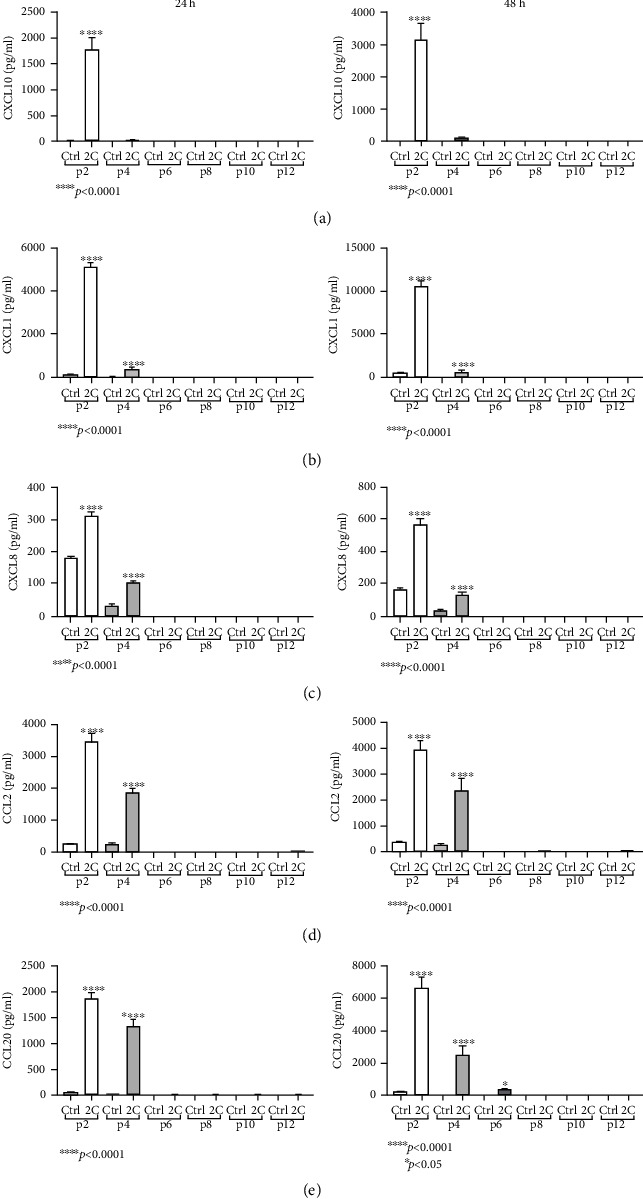
HIOs exhibit mesenchymal and epithelial chemokine protein responses to inflammatory cytokines. IL-1*α* and TNF-*α* (2C) 24 h and 48 h stimulation induced the protein expression of CXCL1 (b), CXCL8 (c), CCL2 (d), and CCL20 (e) in passages 2 and 4, while CXCL10 was induced only in passage 2 (a). CCL20 was also induced in passage 6, after 48 h stimulation. Concentrations of cytokines used: IL-1*α* 5 ng/ml, TNF-*α* 50 ng/ml. All experiments were performed in triplicate. Data are presented as the mean ± standard error of the mean (SEM).

The same pattern was observed in the protein level for chemokines CXCL10, CXCL1, CXCL8, CCL2, and CCL20 (Figure 6). Specifically, in passage 2, stimulation with IL-1*α* and TNF-*α* for 24 and 48 hours greatly upregulated CXCL10 (24 h: 1779 ± 234 pg/ml; 48 h: 3134 ± 305.3 pg/ml; and *p* < 0.0001; Figure 6(a)), CXCL1 (24 h: 5214 ± 113.9 pg/ml; 48 h: 10618 ± 296.2 pg/ml; and *p* < 0.0001; Figure 6(b)), CXCL8 (24 h: 312.5 ± 10.89 pg/ml; 48 h: 571.7 ± 33.9 pg/ml; and *p* < 0.0001; Figure 6(c)), CCL2 (24 h: 3458 ± 237.2 pg/ml; 48 h: 3965 ± 15.54 pg/ml; and *p* < 0.0001; Figure 6(d)), and CCL20 (24 h: 1870 ± 107.7 pg/ml; 48 h: 6669 ± 361.7 pg/ml; and *p* < 0.0001; Figure 6(e)). In passage 4, IL-1*α* and TNF-*α* stimulation also upregulated, but in a less extent, the chemokines CXCL1 (24 h: 356.3 ± 26.1 pg/ml; 48 h: 686.9 ± 30.21 pg/ml; and *p* < 0.0001; Figure 6(b)), CXCL8 (24 h: 103.4 ± 2.1 pg/ml; 48 h: 125 ± 11.3 pg/ml; and *p* < 0.0001; Figure 6(c)), CCL2 (24 h: 1848 ± 135.9 pg/ml; 48 h: 2396 ± 261.4 pg/ml; and *p* < 0.0001; Figure 6(d)), and CCL20 (24 h: 1331 ± 129 pg/ml; 48 h: 2501 ± 317.3 pg/ml; and *p* < 0.0001; Figure 6(e)), while CXCL10 was unaffected. In passage 6, only CCL20 was upregulated (368.7 ± 33.59 pg/ml, *p* < 0.05; Figure 6(e)), after the 48 h IL-1*α* and TNF-*α* stimulation, while all the other chemokines were undetectable. In higher passages, none of the studied chemokines were traceable for both the stimulated and unstimulated organoids, while CXCL11 protein expression was absent in all passages and conditions.

**Figure 7:**
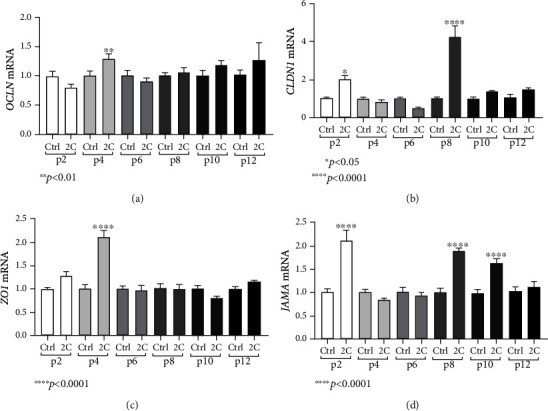
Epithelial responses to proinflammatory cytokines decrease during passaging. IL-1*α* and TNF-*α* (2C) induced the mRNA expression of OCLN in passage 4 (a), CLDN1 in passages 2 and 8 (b), ZO1 in passage 4 (c), and JAMA in passages 2, 8, and 10 (d). Concentrations of cytokines used: IL-1*α* 5 ng/ml, TNF-*α* 50 ng/ml. All experiments were performed in triplicate. The gene expression of each studied gene was normalized against GAPDH gene expression in the same sample using the 2^-*ΔΔ*Ct^ method. In every passage, expression levels of treated organoids were normalized against those of the control ones. Data are presented as the mean ± standard error of the mean (SEM).

In contrast, a different pattern was observed in the expression of tight junction molecules in response to inflammatory stimuli. CLDN1 and JAMA were upregulated in passage 2 in response to IL-1*α* and TNF-*α* (CLDN1: 2.03-fold, ±0.17, and *p* < 0.05; JAMA: 2.10-fold, ±0.23, and *p* < 0.0001, Figures 7(b) and 7(d)), but their expression was later returned to basal levels in passages 4 and 6. In passage 4, only OCLN and ZO1 showed a statistically significant mRNA upregulation that was abolished in later passages (OCLN: 1.28-fold, ±0.087, and *p* < 0.01; ZO1: 2.11-fold, ±0.13, and *p* < 0.0001, Figures 7(a) and 7(c)). In passage 8, only CLDN1 and JAMA were statistically significantly upregulated in response to IL-1*α* and TNF-*α* (CLDN1: 4.27-fold, ±0.55, and *p* < 0.0001; JAMA: 1.91-fold, ±0.04, and *p* < 0.0001, Figures 7(b) and 7(d)), while in passage 10, only JAMA remained upregulated in response to the two proinflammatory cytokines (1.63-fold, ±0.09, and *p* < 0.0001, Figure 7(d)), suggesting that structural molecules of the epithelium retain responsiveness to inflammatory stimuli in late passages despite the loss of mesenchymal responses. Finally, in passage 12, no effect in any studied tight junction molecule was observed, after the IL-1*α* and TNF-*α* stimulation.

## 4. Discussion

In this study, we show that HIOs mature and change through sequential passages, and their mesenchymal component gradually reduces with time. We have also observed that HIOs respond differently to the proinflammatory cytokines, IL-1*α* and TNF-*α*, depending on the passage, suggesting that the gradual loss of the stromal component reflects on the functionality of HIOs. Specifically, we showed that IL-1*α* and TNF-*α* stimulation upregulated the mRNA of various fibrotic and inflammatory factors in early, but not late, passages and this pattern was also observed at the protein level for the inflammatory chemokines CXCL10, CXCL1, CXCL8, CCL2, and CCL20. IL-1*α* and TNF-*α* stimulation had no effect on the HIO structure and growth rate in either the incubation period or the culture passage. In addition, we have also shown that HIOs maintain their structure through serial culture passages, and although their growth rate continues, it is probably attributed to the active proliferation state of their epithelial cells.

Organoids have been described as a more favorable *in vitro* tool for disease modeling for several reasons. Firstly, they can more accurately mimic the tissue architecture of the respective organ. As shown in our study, HIOs resemble the human intestinal tissue as they develop the villi, different types of epithelial cells including goblet and endocrine cells, and supporting stroma. Furthermore, they exhibit both the inflammatory and fibrotic responses to inflammatory stimuli similar to the intestinal tissue. Apart from HIOs, other types of organoids have been developed to accurately simulate different organs, such as lung organoids that are structured into alveolars, airways, and lung buds [20], liver organoids that consisted of hepatocytes and cholangiocytes that form a functional bile canalicular network [21], renal organoids that formed in most cases glomeruli and renal tubules [22], and many others [23]. Secondly, organoids can reduce the need for 2D cultures of primary cells; in most cases, they are difficult to isolate, characterize, and maintain in culture for prolonged periods of time [24]. Thirdly, HIOs contain healthy epithelial cells, which are easily studied and expanded, in contrast to primary epithelial cells which initiate apoptotic processes following isolation [25], and offer a more relevant human physiology model than using cultures of epithelial immortalized cell lines [26]. Fourthly, HIOs enable researchers to carry out high-throughput screening experiments without the need for large numbers of experimental animals (according to the Reduce-Replace-Refine principle). And finally, HIOs provide a more analytical approach by being able to separately study epithelial and mesenchymal responses from immune responses of the intestinal mucosa.

Apart from HIOs, which are pluripotent stem cell-derived organoids, there are also adult stem cell-derived 3D structures, called enteroids. Enteroids can be developed from isolated adult Lgr5^+^ stem cells or intestinal crypts containing these cells [27], using a more simplified and easier method than HIO development [28], and since they are developed from a less potent stem cell population, they are easier to maintain and culture [27]. Nonetheless, the main disadvantage of enteroids is that they only consist of epithelial cells, lacking the mesenchymal component [29], making HIOs the model of choice in studies investigating epithelial and mesenchymal interactions.

We showed that the mesenchymal component of HIOs, although gradually decreased over culture time, plays a significant role in both the inflammatory and fibrotic responses to proinflammatory stimuli, suggesting the importance of mesenchymal cells in organoid functional studies. Indeed, previous works from our group and others have highlighted the importance of mesenchymal cells in chronic intestinal inflammation and fibrosis. We have shown that intestinal subepithelial myofibroblasts (SEMFs) express various interleukin receptors, and stimulation with different Th-related cytokines leads to different fibrotic responses from SEMFs [9]. In this study, we concluded that the reduced responsiveness to IL-1*α* and TNF-*α* is possibly due to the mesenchymal component reduction and the consequent decrease of epithelial-mesenchymal crosstalk. We have previously shown that SEMFs interact with epithelial cells, as supernatants from previously stimulated HT-29 epithelial cells induce the expression of both the fibrotic and proinflammatory molecules, such as collagen and TL1A, respectively [30, 31]. Others have previously shown that high Oncostatin M (OSM) expression in patients with IBD is associated with failure to anti-TNF therapy and that high expression of its receptor is found in intestinal stromal cells, suggesting that mesenchymal cells have a significant role in IBD patient heterogeny to respond to anti-TNF agents [2]. SEMF-dependent IBD patient heterogeny is also highlighted in the research by Beswick et al. They showed that SEMFs isolated from inflamed intestinal regions of UC patients have a stronger capacity to suppress Th1 cell activity than CD or healthy SEMFs, as they overexpress programmed cell death protein 1 (PD-1), a molecule implicated in the regulation of Th immune responses [32]. In a recent study, Toll-like receptor 4 (TLR4) depletion in CCD-18Co cells, an intestinal fibroblast cell line, resulted in increased matrix metalloproteinase-1 (MMP-1) and decreased tissue inhibitor of metalloproteinase (TIMP) and collagen *α*1 expression [33], suggesting that innate immune responses directly regulate the fibrotic phenotype of SEMFs. More recent studies have also shown that the fibrotic phenotype of SEMFs depends on the tissue stiffness, as ileum isolated CD SEMFs have upregulated levels of the collagen crosslinking enzyme lysyl oxidase and lead to high ECM contraction [34], and this may be regulated through endoplasmic reticulum stress-related gene overexpression [35].

In this study, we showed that early-passage HIOs overexpress fibrotic factors in response to inflammatory stimuli. In the same notion, Rodansky et al. were the first to show that HIOs are a promising fibrotic model, as HIOs overexpress several fibrotic factors in response to a dose-dependent TGF-*β* stimulation [36]. In a more recent study by the same research group, Steiner et al. utilized HIOs as a fibrotic model to prove that the inhibition of AXL, a receptor tyrosine kinase, could impede the TGF-*β*1-induced fibrotic overexpression [37]. Apart from the fibrotic responses, we have also shown that several chemokines are greatly overexpressed, at the mRNA and protein levels, when early-passage HIOs are stimulated with IL-1*α* and TNF-*α*. Other studies using induced pluripotent stem cell- (iPSC-) derived intestinal organoids as an *in vitro* inflammation model have reported similar results. Karve et al. observed that iPSC-derived intestinal organoids infected with a pathogenic strain of *Escherichia coli* produced elevated levels of IL-8 (CXCL8) and IL-18 [38]. Workman et al. showed that iPSC-derived intestinal organoids overexpressed the chemokines CXCL9, CXCL10, and CXCL11 in response to IFN-*γ* stimulation [39]. Finally, Onozato et al. reported that TNF-*α* induces the upregulation of TNF-*α* and IL-1*β* and abolishes the expression of Chromogranin A in iPSC-derived intestinal organoids. When TNF-*α* was combined with TGF-*β*, iPSC-derived intestinal organoids produced high levels of the profibrotic molecules, *α*-SMA, vimentin, collagen type I, and fibronectin, and the proinflammatory factors, TNF-*α* and IL-1*β* [40], suggesting that this is a promising model for studying inflammatory and fibrotic responses.

Overall, the novelty of our study lies in the fact that we show that there is a gradual downregulation of several fibrotic and mesenchymal markers, as HIOs progress from passage to passage, and there are different responses to proinflammatory cytokines depending on the passage. Other recent studies have also shown that organoids continue to mature and change throughout their culture, mimicking the process of embryonic to fetal and adult development [13–15]. Our results are in agreement with these studies and further verify the phenomenon of organoid maturation at late passages.

## 5. Conclusions

In conclusion, we show that embryonic stem cell-derived HIOs are supported by a mesenchymal component, which is gradually reduced over sequential passages. This mesenchymal component plays a significant role in both the epithelial and mesenchymal cell inflammatory and fibrotic responses, and its reduction leads to loss of functionality, as well as unresponsiveness to proinflammatory stimuli. Therefore, inflammatory and fibrotic studies employing HIOs should be focused on early passages. Further studies are needed to elucidate the mechanisms of HIO transformation and to identify the molecular pathways that are implicated in HIO maturation.

## Figures and Tables

**Table 1:** Gene-specific primers used in real-time PCR.

Gene	Forward	Reverse	Reference
GAPDH	GACATCAAGAAGGTGGTGAA	TGTCATACCAGGAAATGAGC	[9]
Collagen type I	CCCTGGAAAGAATGGAGATGAT	ACTGAAACCTCTGTGTCCCTTCA	
Collagen type III	GCTCTGCTTCATCCCACTATTA	TGCGAGTCCTCCTACTGCTAC	
Fibronectin	CCAGTCCACAGCTATTCCTG	ACAACCACGGATGAGCTG	
*α*-SMA	AATGCAGAAGGAGATCACGG	TCCTGTTTGCTGATCCACATC	
TF	TTCAGTGTTCAAGCAGTGATTCC	ATGATGACCACAAATACCACAGC	
CD90	CGCTCTCCTGCTAACAGTCTT	CAGGCTGAACTCGTACTGGA	[41]
CCL2	AGGAAGATCTCAGTGCAGAGG	AGTCTTCGGAGTTTGGGTTTG	[42]
CCL20	GCTGCTTTGATGTCAGTGC	GCAGTCAAAGTTGCTTGCTTC	[43]
CXCL1	GCCCAAACCGAAGTCATAGCC	ATCCGCCAGCCTCTATCACA	[44]
CXCL8	TGGGTGCAGAGGGTTGTG	CAGACTAGGGTTGCCAGATTTA	[42]
CXCL10	CCTGCTTCAAATATTTCCCT	CCTTCCTGTATGTGTTTGGA	
CXCL11	GACGCTGTCTTTGCATAGGC	GGATTTAGGCATCGTTGTCCTTT	[45]
CLDN1	CGATGCTTTCTGTGGCTAA	AGTGGCTGACTTTCCTTGT	[46]
OCLN	CCTATAAATCCACGCCGGTTC	TCAAAGTTACCACCGCTGCTG	
ZO1	AACAGCCCTACCCATCTCG	CGTGGAAAGTACCCTCGTT	
JAMA	CGAGAGGAAACTGTTGTGCC	AACGAGTCTGGTGGTGTCTC	[47]

## Data Availability

The data used to support the findings of this study are available from the corresponding author upon request.

## Supplementary Materials

Supplementary Materials Supplementary Figure 1: HIO growth rate in each passage and Ki67 proliferation marker staining. (A) Representative snapshots at culture days 0 and 10 in each passage, showing that the HIO intestinal structure remained stable. Magnification was set at 4x. (B) Semiquantitative calculation of the percentage of the organoid growth rate that shows an increase in diameter from day 0 to day 10, suggesting that HIOs continue to mature their luminal structures even in late passages. (C) Double staining for the expression of Ki67, a well-known proliferation marker, and EpCam, an epithelial marker, in a late-passage organoid. Ki67-positive expression is found in HIO epithelial cells. Representative 40x immunofluorescence snapshots are shown in (C). Supplementary Figure 2: characterization of the main developmental stages prior to HIO formation. (A) H1 pluripotent stem cell line stained against the embryonic stem cell markers, Nanog, SOX2, and OCT4. (B) Definitive Endoderm stained against SOX17 and FOXA2, two transcription factors required for the development of the definitive gut endoderm and the intestinal tissue, respectively. (C) Mid-/Hindgut spheroids stained against CDX2, an intestinal epithelial marker, and vimentin and E-cadherin, mesenchymal and epithelial markers, respectively. Representative 40x immunofluorescence snapshots are shown in (A–C). Supplementary Figure 3: IL-1*α* and TNF-*α* stimulation does not affect the HIO structure and growth rate. Representative snapshots of unstimulated (Ctrl) and IL-1*α* and TNF-*α* (2C)-stimulated HIOs in passages 2 (A and B), 6 (C and D), and 12 (E and F), showing no changes in the growth rate and structure. Magnification was set at 4x.
