# Supplementary material for: Development of a Human Intestinal Organoid Model for In Vitro Studies on Gut Inflammation and Fibrosis
Source: Stem Cells Int. 2021 Jul 27;2021:9929461. doi: 10.1155/2021/9929461 (PMC8331310; doi:10.1155/2021/9929461)
Supplement: Supplementary Materials — Supplementary Figure 1: HIO growth rate in each passage and Ki67 proliferation marker staining. (A) Representative snapshots at culture days 0 and 10 in each passage, showing that the HIO intestinal structure remained stable. Magnification was set at 4x. (B) Semiquantitative calculation of the percentage of the organoid growth rate that shows an increase in diameter from day 0 to day 10, suggesting that HIOs continue to mature their luminal structures even in late passages. (C) Double staining for the expression of Ki67, a well-known proliferation marker, and EpCam, an epithelial marker, in a late-passage organoid. Ki67-positive expression is found in HIO epithelial cells. Representative 40x immunofluorescence snapshots are shown in (C). Supplementary Figure 2: characterization of the main developmental stages prior to HIO formation. (A) H1 pluripotent stem cell line stained against the embryonic stem cell markers, Nanog, SOX2, and OCT4. (B) Definitive Endoderm stained against SOX17 and FOXA2, two transcription factors required for the development of the definitive gut endoderm and the intestinal tissue, respectively. (C) Mid-/Hindgut spheroids stained against CDX2, an intestinal epithelial marker, and vimentin and E-cadherin, mesenchymal and epithelial markers, respectively. Representative 40x immunofluorescence snapshots are shown in (A–C). Supplementary Figure 3: IL-1α and TNF-α stimulation does not affect the HIO structure and growth rate. Representative snapshots of unstimulated (Ctrl) and IL-1α and TNF-α (2C)-stimulated HIOs in passages 2 (A and B), 6 (C and D), and 12 (E and F), showing no changes in the growth rate and structure. Magnification was set at 4x. [file 9929461.f1.zip › 9929461.f3.docx]

**Supplementary Figure 3**

**
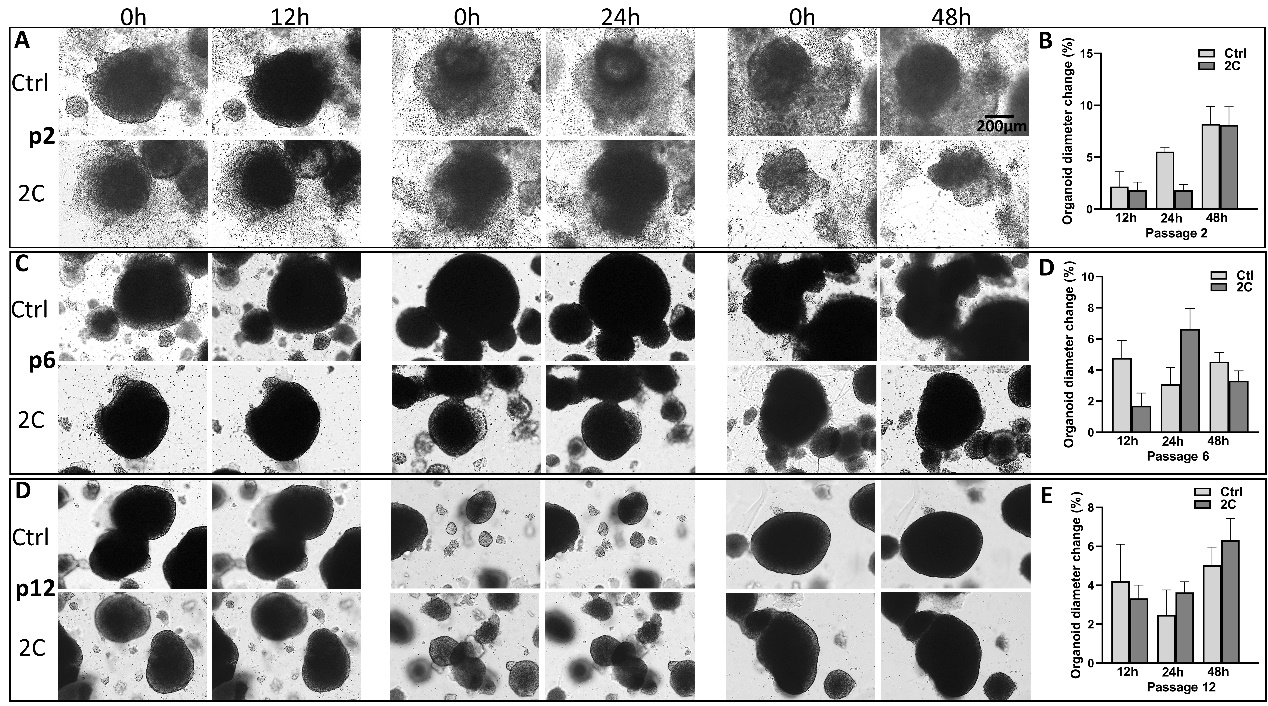
**

**Supplementary Figure 3 Legend**

**Supplementary Figure 3.** IL-1α and TNF-α stimulation does not affect the HIOs structure and growth rate. Representative snapshots of unstimulated (Ctrl) and IL-1α and TNF-α (2C) stimulated HIOs in passages 2 (A and B), 6 (C and D) and 12 (E and F) showing no changes in growth rate and structure. Magnification was set at 4x.
